# Effects of centrifugation-based therapeutic plasma exchange on coagulation parameters in dogs with canine visceral leishmaniasis

**DOI:** 10.1080/01652176.2026.2706032

**Published:** 2026-10-14

**Authors:** Suellen Rodrigues Maia, Silvano Salgueiro Geraldes, Fábio dos Santos Nogueira, Maria Gabriela Picelli de Azevedo, Reiner Silveira de Moraes, Julia Franco Ferreira, Teresa Alessandra de Lima Núñez, Regina Kiomi Takahira, Alessandra Melchert, Adriano Sakai Okamoto, Priscylla Tatiana Chalfun Guimarães-Okamoto

**Affiliations:** a Department of Veterinary Clinics, School of Veterinary Medicine and Animal Science, São Paulo State University (UNESP), Botucatu, São Paulo, Brazil; b Department of Veterinary Medicine, Federal Institute of South Minas, Muzambinho, Minas Gerais, Brazil

**Keywords:** Apheresis, dog, coagulopathy, bleeding, thrombosis

## Abstract

As in humans, therapeutic plasma exchange (TPE) has been investigated in veterinary medicine for managing immune-mediated diseases such as canine visceral leishmaniasis (CVL). TPE may improve clinical outcomes by removing circulating immune complexes and excess antibodies; however, its physiological effects must be understood to ensure safety. Given the scarcity of studies in animals, this study evaluated the effects of centrifugation-based TPE in dogs with CVL receiving fresh frozen plasma (FFP) as replacement fluid. Thirty-one dogs with CVL that failed to respond to conventional therapy were allocated to two groups: clinical treatment alone (*n* = 18) and clinical treatment plus TPE (*n* = 13). The TPE group underwent one daily session for three consecutive days. Coagulation profiles were assessed before and after each session. A progressive decrease in platelet counts occurred across sessions, but without significant thrombocytopenia or critical alterations in activated partial thromboplastin time (aPTT) or prothrombin time (PT), which remained within reference ranges. Transient hypocalcemia occurred after the first session and was promptly corrected with calcium gluconate supplementation. Fibrinogen levels decreased mildly without clinical evidence of bleeding. Clinical complications were minimal and effectively managed. In conclusion, TPE was well tolerated, producing predominantly transient and controllable effects on hemostasis and overall clinical homeostasis under the conditions of this study.

## 1. Introduction

Among the various apheresis modalities, therapeutic plasma exchange (TPE) involves the separation, removal, and subsequent replacement of a patient’s plasma through an extracorporeal circuit (Schwartz et al. 2013; Reeves and Winters 2014). The indication for this therapeutic approach applies to conditions in which the removal of high–molecular-weight plasma substances—such as autoantibodies, alloantibodies, immune complexes, endotoxins, and toxins—may improve the response to conventional treatment or provide temporary clinical stabilisation (Miller 1992; Khorzad et al. 2011; Ward, 2011; Kaplan 2013; Walton et al. 2017a, 2017b, Alamartine and Maillard 2019).

The removal of target substances by TPE can be achieved via centrifugation or membrane filtration (Reeves and Winters 2014; Alamartine and Maillard 2019). Both techniques enable the elimination of large molecules (>15,000 daltons). However, while centrifugation separates components on the basis of density through gravitational force, membrane filtration depends on molecular size, which is determined by the pore diameter of the filtration membrane (Gurland et al. 1984; Ward 2011; Kaplan 2013; Williams and Balogun 2014). Regardless of the technique used, TPE is a nonselective procedure that removes both pathological and physiological plasma components, requiring the simultaneous use of replacement fluids such as fresh frozen plasma (FFP) or human serum albumin to maintain circulatory stability, although synthetic colloids and crystalloids may also be used (Kaplan 2013; de Back et al. 2019).

Although maintenance of blood volume and colloid osmotic pressure during TPE can be achieved through the combined infusion of colloid and crystalloid solutions, haemostatic impairment may still occur if FFP is not used or is administered in insufficient quantities (Zantek et al. 2019). Particular attention is required in protocols involving repeated sessions in a short period (Zrimšek and Pernat 2021). In such settings, prolonged coagulation times resulting from the removal of coagulation factors and regulatory proteins, along with concomitant platelet reduction and the anticoagulant effect of citrate used during the session, may increase the risk of haemorrhagic or thrombotic events (Zantek et al. 2019).

TPE has already been well established and is continuously evolving in human medicine (Rock 2019). TPE is applied to a wide range of immune and nonimmune conditions (Von Baeyer 2003; Chegini et al. 2019; Fernández-Zarzoso et al. 2019; Padmanabhan et al. 2019) and is generally considered a safe procedure (Pham et al. 2019).

In veterinary medicine, however, TPE remains an emerging and relatively underreported therapy, mainly because of its high cost and the need for specialised equipment and trained personnel (Khorzad et al. 2011; Francey and Schweighauser 2019). Its application has been described in several immune-mediated (Matus et al. 1985; Crump and Seshadri 2009; Perondi et al. 2018; Kopecny et al. 2020) and systemic conditions (Matus et al. 1983; Bartges et al. 1990; Skulberg et al. 2018; Lippi et al. 2015; Walton et al. 2017a, 2017b), supporting its potential as an adjunctive therapeutic tool.

Many of these conditions share a common immunopathological basis, characterised by excessive production of globulins, antibodies, autoantibodies, and alloantibodies, as well as the formation and deposition of immune complexes (Pedersen 1999). In this context, canine visceral leishmaniasis (CVL) serves as a relevant example, as it is a parasitic disease in which host immune defenses may trigger variable cytotoxic and humoral responses. In some animals, a predominance of the humoral response leads to excessive production of nonprotective antibodies, which is associated with disease severity and progression (Pinelli et al. 1994; Barbiéri 2006).

Considering the characteristics of TPE, its application in dogs with CVL represents a promising therapeutic perspective. However, in veterinary medicine, this approach still requires further investigation regarding its delivery, safety, and physiological effects. Given the limited understanding of TPE’s impact on coagulation parameters in veterinary patients and the hypothesis that the procedure may influence the risk of coagulopathies, the present study aimed to describe the effects of centrifugation-based TPE in dogs with CVL receiving FFP as part of the replacement fluid and citrate as the extracorporeal anticoagulant, emphasising potential complications and the establishment of safe therapeutic protocols.

## 2. Materials and methods

The study was conducted at the Dialysis Centre of the Veterinary Teaching Hospital, Faculty of Veterinary Medicine and Animal Science, São Paulo State University (FMVZ-UNESP), Botucatu, Brazil, within the Canine and Feline Nephrology and Urology Service. The research was carried out in partnership with the Mundo Animal Veterinary Hospital (Andradina, SP, Brazil) and Professor Fábio dos Santos Nogueira, DVM, MSc, PhD, from the Faculty of Agrarian Sciences of Andradina—Andradina Educational Foundation (SP, Brazil), who was responsible for transporting the animals to the Dialysis Centre at UNESP–Botucatu. After each session, the animals returned to the referring clinic, where their clinical treatment was continued. The research protocol was reviewed and approved by the Institutional Animal Care and Use Committee (CEUA) of São Paulo State University (protocol no. 0193/2021). The inclusion of the animals was performed with the informed consent of their owners, who were fully informed about the procedures and voluntarily signed a written consent form.

### 2.1. Animals

#### 2.1.1. Selection criteria

Dogs of different breeds and both sexes, aged over one year, were included in the study. The selection was based on the diagnosis of CVL in animals that failed to respond to conventional immunomodulatory therapy, characterised by clinical and laboratory progression, worsening, or refractoriness associated with excessive globulin and antibody production. The clinical presentation of the animals was consistent with forms of CVL commonly described in the LeishVet guidelines proposed by Solano-Gallego et al. (2011). Some animals also exhibited clinical manifestations related with immune-mediated or immune complex deposition, such as polyarthritis, synovitis, polymyositis, osteomyelitis, uveitis, vasculitis, or choroid plexus deposition.

The diagnosis of CVL was established using standardised diagnostic methods applied to all included animals. For the serological assessment of canine visceral leishmaniasis (CVL), a commercial rapid ELISA test, SNAP Leishmania® (IDEXX Laboratories Inc., Westbrook, ME, USA), was used, which is based on the detection of anti-*Leishmania infantum* antibodies (90–98% sensitivity and 95–99% specificity). Additionally, all animals underwent direct parasitological evaluation through bone marrow and/or lymph node aspirates for the identification of amastigote forms. The combination of serological and parasitological methods was used to support the diagnosis and confirm active infection.

#### 2.1.2. Exclusion criteria

Dogs diagnosed with CVL but presenting definitive or presumptive evidence of specific coagulation abnormalities such as haemophilia, vitamin K antagonist intoxication, or von Willebrand disease were excluded. These conditions were investigated only in animals with clinical signs or a history suggestive of coagulopathy. Additionally, animals presenting with shock or severe hemodynamic instability, as well as those testing positive for ehrlichiosis, babesiosis, anaplasmosis, or dirofilariasis (SNAP 4Dx Plus® -IDEXX Laboratories Inc., Westbrook, ME, EUA) (Dokuzeylül et al. 2024), were excluded from the study.

### 2.2. Experimental groups

The animals were assigned to two experimental groups: the clinical treatment group (GCT) and the clinical treatment plus therapeutic plasma exchange group (GTPE). Each dog in the GTPE underwent one TPE session per day for three consecutive days (1, 2 and 3), with each session representing one moment.

### 2.3. Medical treatment

Following CVL diagnosis, specific antileishmanial therapy was initiated, and patients were closely monitored. The treatment protocol included the leishmanicidal drug miltefosine (2 mg/kg orally) and the leishmaniostatic agent allopurinol (10 mg/kg orally), which were administered according to standardised intervals and durations. In addition, all dogs wore repellent collars containing deltamethrin. Immunotherapy and immunomodulatory, supportive, and symptomatic treatments were provided as needed and tailored to each patient’s clinical condition.

### 2.4. Therapeutic Plasma Exchange (TPE) by centrifugation

#### 2.4.1. Vascular access

All animals in the GTPE underwent central venous catheterisation via a modified Seldinger technique (Seldinger 1953; Taylor and Palagiri 2007). A double-lumen catheter was inserted into the right jugular vein, with the distal tip positioned at the junction between the cranial vena cava and the right atrium. Depending on the animal’s size, either an 8 Fr × 12.5 cm (Joline®) or an 11 Fr × 20 cm (Joline®) catheter was used. Immediately after jugular catheterisation, a radiographic examination was performed to confirm the correct positioning of the catheter.

#### 2.4.2. Technique

TPE sessions were performed via a Cobe Spectra® machine (Terumo BCT, Colorado, USA), which operates via centrifugation. In this system, blood is continuously withdrawn from the patient, centrifuged, and separated (red blood cells, plasma, platelets, and leucocytes) by density. The plasma, as the target component, is removed and simultaneously replaced—that is, as the patient’s plasma is withdrawn, a replacement solution is infused.

The replacement solution consisted partly of saline (0.9% NaCl) and partly of fresh frozen plasma (FFP), with FFP comprising at least 50% of the total replacement volume. One TPE session per day was performed for three consecutive days (1, 2, and 3) for all dogs in the TPE group. Each session was configured to ensure that at least one plasma volume exchange was achieved. Diphenhydramine (2 mg/kg, SC) and hydrocortisone (50 mg/kg, slow IV) were administered 30 minutes prior to each session. The use of corticosteroids in combination with antihistamines was intended to reduce the risk of transfusion reactions, as described in some apheresis protocols for both humans and animals (Kaplan 2013; Francey et al. 2021).

Considering each animal’s blood volume, the extracorporeal circuit (285 mL) was initially primed with either 0.9% NaCl solution or packed red blood cells (PRBCs) to prevent initial hypotension during the session. System anticoagulation was achieved via a citrate-containing bag connected to the circuit, maintaining a ratio of 1:10 anticoagulant to blood. Continuous infusion of 10% calcium gluconate (1 mL/kg/h) was administered throughout the procedure to minimise citrate-induced hypocalcemia.

The total blood volume (TBV) was calculated according to the following formula: TBV = body weight (kg) × 0.08. Haematic volume (HV) was obtained via the following formula: HV = TBV (mL) × haematocrit (%). The patient’s plasma volume (PV) was then calculated as:

During the procedure, the dogs were physically restrained on a stainless-steel table without the need for sedation.

### 2.5. Clinical examination

All dogs underwent physical and clinical parameter assessments before, during, and after the procedure. The following parameters were recorded: systolic blood pressure (SBP) via a Parks 811-B vascular Doppler, rectal temperature (RT), heart rate (HR), and respiratory rate (RR). In addition, data from both general and specific physical examinations related to the presentation of CVL were collected for all enroled animals.

### 2.6. Biological sample collection and laboratory evaluations

Blood samples were obtained from all dogs via jugular venipuncture. In the GTPE, samples were collected immediately before and after each TPE session to allow intraindividual comparisons of procedure-related effects, whereas in the GCT, sampling was performed only at the time of inclusion, prior to the initiation of therapy, to provide reference values for between-group comparisons.

A portion of each sample was immediately placed into sterile tubes containing 10% EDTA anticoagulant (BD Vacutainer®, New Jersey, USA) for complete blood count (CBC) analysis. Another portion was collected in citrate-containing tubes for coagulation assays, including prothrombin time (PT), activated partial thromboplastin time (aPTT), and fibrinogen concentration, via a Mono Canal Clotimer® coagulometer. Blood gas analysis was also performed to determine the blood pH, ionised calcium, sodium bicarbonate, and potassium levels.

### 2.7. Statistical analysis

Sample size determination was performed via SigmaPlot v.11 (Systat Software, Inc., 2008), achieving a test power > 0.80 on the basis of the most relevant variables (red blood cell count, haemoglobin, platelet count, and leucocyte count), with only 13 samples and *α* = 0.05. The main differences and standard deviations were calculated according to a pilot study previously published in a high-impact journal (Maia et al. 2025).

For statistical analysis, the Shapiro–Wilk test was used to assess the normality of quantitative data. For each variable, comparisons were made between each time point in the TPE group and the control group measurement (obtained at a single time point). In addition, pre- and post-procedure measurements were compared within the TPE group.

For normally distributed variables, differences between two groups were analysed via Student’s *t*-test, whereas for nonnormally distributed variables, the Mann–Whitney *U* test was applied. For normally distributed variables with repeated measures, the paired *t*-test was used; otherwise, the Wilcoxon signed-rank test was employed.

For better visualisation of these analyses, bar graphs representing the mean ± standard deviation (parametric approach) and boxplots (nonparametric approach) were generated. The level of statistical significance was set at 5% (*p* < 0.05), and all analyses were conducted in R software version 4.1.0 (R Core Team 2021).

## 3. Results

Thirty-one dogs were included in the study, of which 18 composed the clinical treatment group (GCT) and 13 the therapeutic plasma exchange group (GTPE). All selected animals had CVL as their underlying diagnosis and, according to the LeishVet guidelines (Solano-Gallego et al. 2011), most dogs included in the present study were in advanced stages of CVL, considering the presence of immune complex–associated manifestations, as well as refractoriness to conventional therapy.

The GCT included 12 males and 6 females, with a mean age of 3.88 ± 2.35 years and a mean body weight of 5.85 ± 0.95 kg, all belonging to the Poodle breed. The GTPE consisted of 7 males and 6 females, with a mean age of 5.48 ± 2.35 years and a mean body weight of 12.21 ± 7.18 kg. This group included five mixed-breed dogs, three Poodles, one Boxer, one Pinscher, one Labrador, one Dachshund, and one Border Collie. The mean age was 3.89 ± 0.32 years in the GCT group and 5.31 ± 2.06 years in the GTPE, with a significant difference between the groups (*p* = 0.013). In the evaluation of physical parameters and blood glucose concentrations in each time point, represented by one session each, comparisons between pre- and post-TPE time points within the GTPE (Table 1) revealed a significant reduction in heart rate (HR) after TPE during the second treatment session. Similarly, systolic blood pressure (SBP) values were significantly lower at the post-TPE time point across all three treatment sessions. Conversely, blood glucose levels significantly increased after TPE, but the difference was statistically significant only in the third session. Despite these changes, all assessed physical parameters remained within the reference range for the species.

**Table 1.** Descriptive statistics and comparative analysis of physical examination variables according to pre- and post-procedure time points in the TPE group (GTPE).

		Pre				Post				
Variable	Moment session	M	MD	SD	IQR	M	MD	SD	IQR	p
Weight	1	12.2	10.2	7.51	9.85	12.23	10.5	7.33	9.95	0.754*
	2	12.27	10.5	7.38	9.7	12.28	10.5	7.27	9.9	0.844*
	3	12.63	10.62	7.75	10.11	12.59	10.95	7.53	9.92	0.562*
HR	1	149.54	148	27.4	44	139.85	140	26.11	42	0.337**
	2	146.77	140	35.04	36	128.54	128	28.77	51	**0.047** **
	3	149.42	164	33.73	47.25	139.17	134	23.75	22.5	0.262**
RR	1	28.77	28	10.63	8	29.54	24	13.02	8	0.944*
	2	30.31	28	11.71	14	29.62	28	10.45	11	0.919*
	3	31	28	12.78	8	23.83	23	9.51	8	0.082*
T(°C)	1	38.73	38.8	0.8	0.5	38.08	38.15	0.61	0.15	0.004*
	2	38.29	38.1	0.58	0.7	37.95	38.1	0.33	0.4	0.068*
	3	38.47	38.45	0.42	0.32	38.09	37.9	0.66	0.38	0.056*
SBP	1	141.62	132	31.61	28	115.46	115	23.71	28	0.007*
	2	132.46	132	15.6	16	114	118	19.63	16	0.021*
	3	145.08	142	25.44	22.5	121.08	115.5	18.37	23	0.017*
Blood glucose	1	113.62	117	15.29	22	149.11	129	41.52	66	0.055*
	2	109.23	108	14.06	14	126.25	126	25.51	32.75	0.158*
	3	110.67	110.5	11.34	12.25	137.17	132.5	29.53	36.25	**0.015** *

^*^ Wilcoxon test.

^**^ Paired t-test; M = mean; MD = median; SD = standard deviation; IQR = interquartile range. HR (heart rate); RR (respiratory rate); T (temperature) and SBP (systolic blood pressure).

In addition to the physical parameters described above, dogs undergoing TPE were also monitored for potential complications such as, hypovolemia, hypotension and hemodilution or adverse events during and after each session such as angioedema, neurological signs (nystagmus) and emesis. Only one animal exhibited two episodes of emesis during the first TPE session, which were likely associated with hypocalcemia, as confirmed by blood gas analysis performed during the procedure. The condition was promptly corrected upon identification. During the second session, the same animal presented hyperthermia and lethargy, and during the final session, a single episode of marked haematuria occurred. Following this event, hydrocortisone (50 mg/kg IV) was administered as a preventive measure for a possible transfusion reaction (Harrell and Kristensen 1995; Maia et al. 2025). The animal remained stable and clinically well during subsequent monitoring until discharge.

Minor complications related to vascular access pressure were observed in a few animals, which were attributed either to patient positioning or to small clots within the catheter. Both situations were easily resolved without compromising the continuity of the sessions. Bilateral chemosis was also observed at the end of the last session in one dog; this same animal presented hypocalcemia after the sessions despite continuous intravenous infusion of 10% calcium gluconate throughout the procedure. This patient was managed with an additional administration of 10% calcium gluconate at the end of each session.

With respect to the erythrogram parameters, the GTPE presented significantly lower red blood cell count, haemoglobin concentrations, and haematocrit values across all evaluation points than did the GCT. However, no statistically significant differences were detected between the time points immediately before and after each TPE session within the GTPE. Information on the MCV, MCHC, RDW, total protein, and metarubricytes in each session at the pre- and pos-time points in the TPE group can be found in Supplementary Table 1.

A significantly lower platelet count was observed after the second and third TPE sessions in the GTPE group than did the GCT group. Compared with the pre-TPE values, the leukogram results revealed a significant increase in total leucocytes and segmented neutrophils after each TPE session. This increase also affected the results of the intergroup comparisons, revealing higher total leucocyte and segmented neutrophil counts after TPE in GTPE patients than in GCT patients. Conversely, lymphocyte counts were significantly lower in the GTPE group than in the GCT group at all evaluated time points. Furthermore, a significant post-procedure decrease in lymphocytes was observed from the second TPE session onward (Figure 1 and Supplementary Table 2).

**Figure 1.**
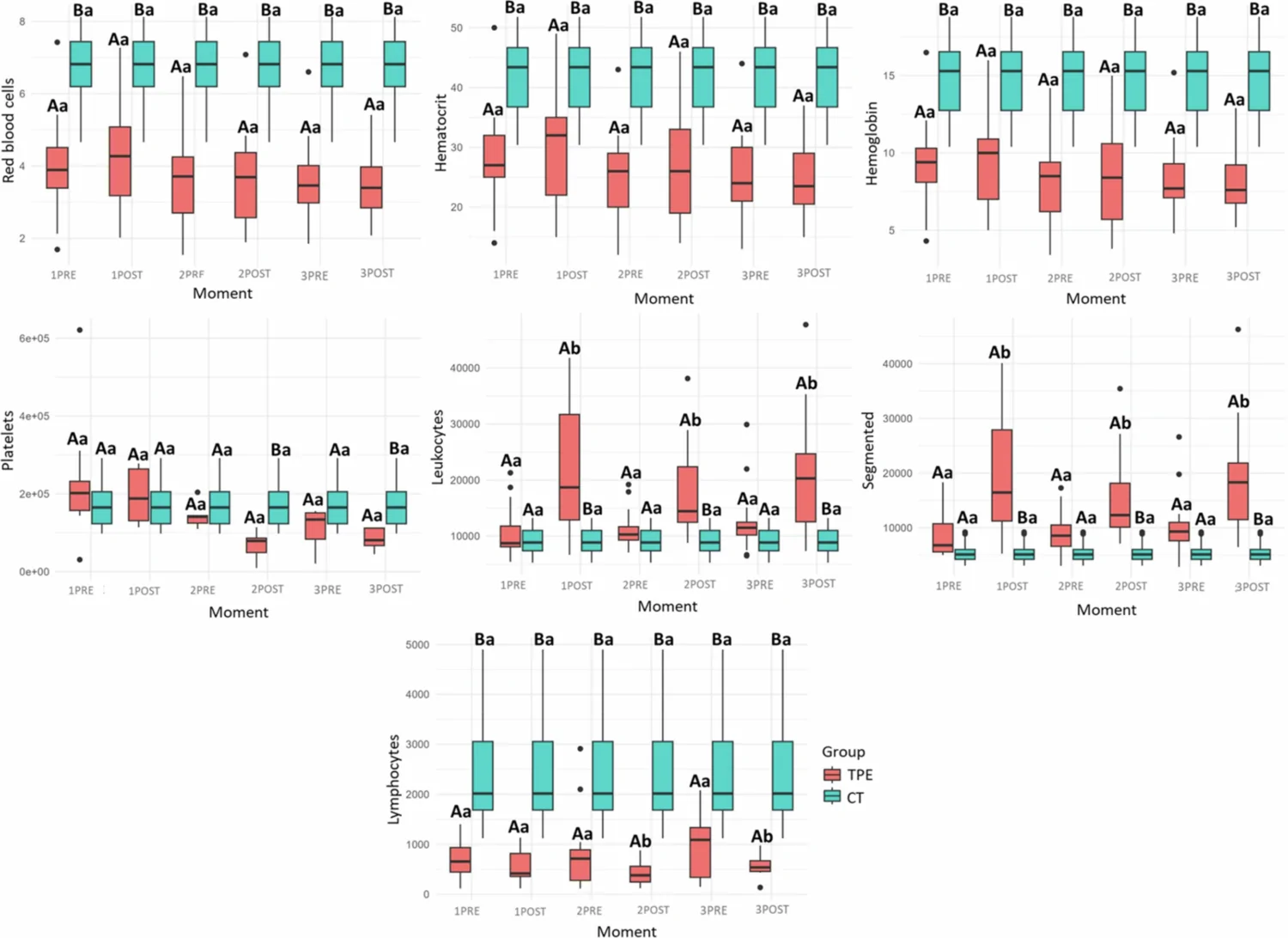
Boxplots of complete blood count variables according to group and time point. Different uppercase letters indicate significant differences between groups at each time point; different lowercase letters indicate significant differences between pre- and post-TPE time points within each session in the GTPE. No comparisons were made between different sessions (1, 2, and 3).

With respect to the blood gas analysis results, significantly lower values of pH, base deficit, and sodium bicarbonate were observed in the GTPE group than in the GCT group at the post-TPE time point. Moreover, within the GTPE, significant reductions in these variables were detected after all the sessions performed. Similarly, chloride levels were significantly lower in the GTPE than in the GCT at all evaluations, showing a marked post-TPE decrease during the first and second sessions (Figure 2 and Supplementary Table 3).

**Figure 2.**
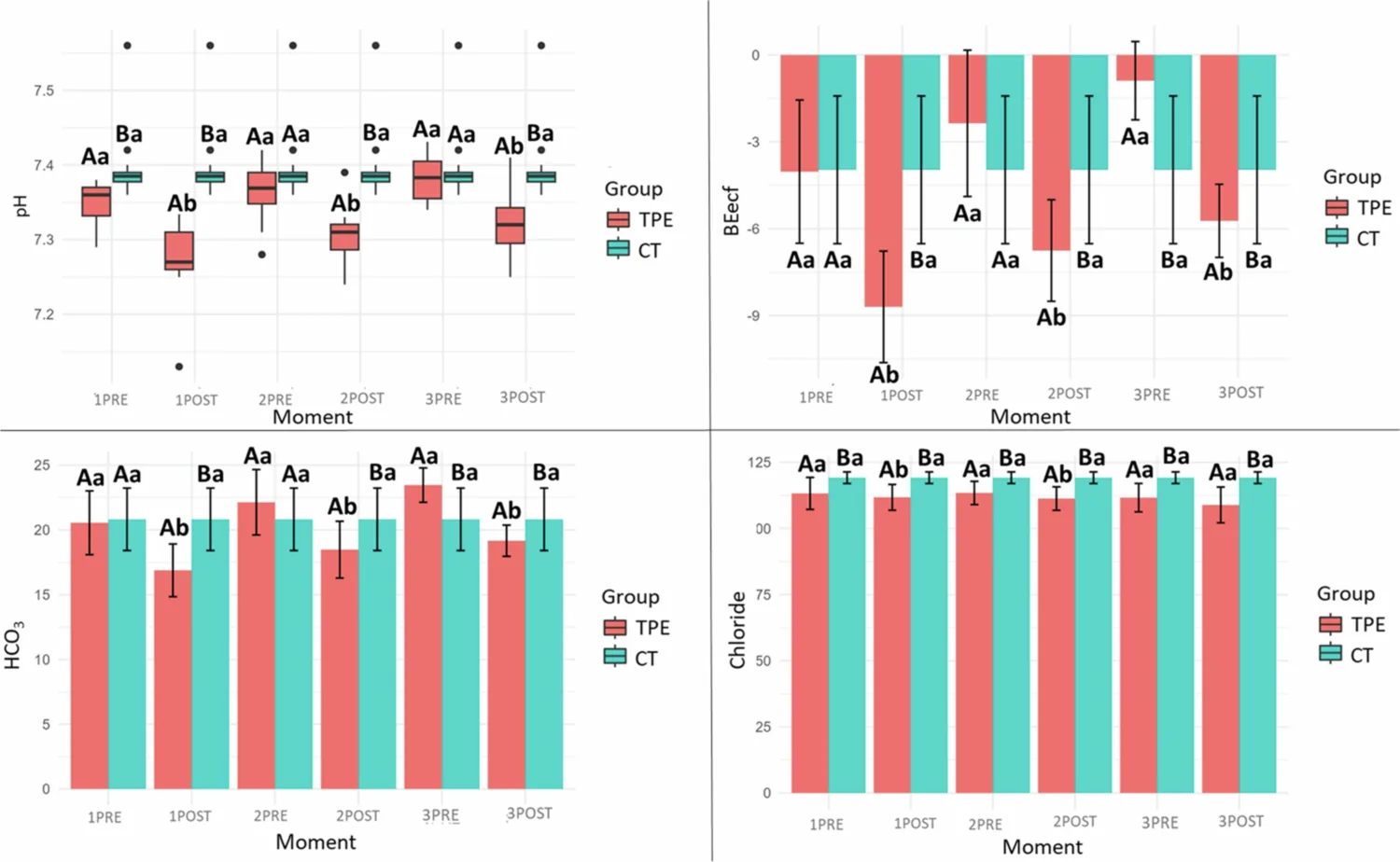
Boxplots or bar graphs of pH, base deficit (BEecf) (average), sodium bicarbonate (HCO_3_) (average), and chloride levels (average) according to group and time point. Different uppercase letters indicate significant differences between groups at each time point; different lowercase letters indicate significant differences between pre- and post-TPE time points within each session in the GTPE. No comparisons were made between different sessions (1, 2, and 3).

A significant reduction in potassium levels was also observed at the post-TPE time point across all sessions in the GTPE, resulting in significantly lower potassium concentrations in the GTPE than in the GCT. Regarding ionised calcium (iCa) assessments, a significant decrease in this electrolyte was detected only at the post-TPE time point of the first session, yielding a significantly lower value than the GCT at that time point. Finally, lactate levels in the GTPE group were significantly higher than those in the GCT group at both time points of the first session (Figure 3).

**Figure 3.**
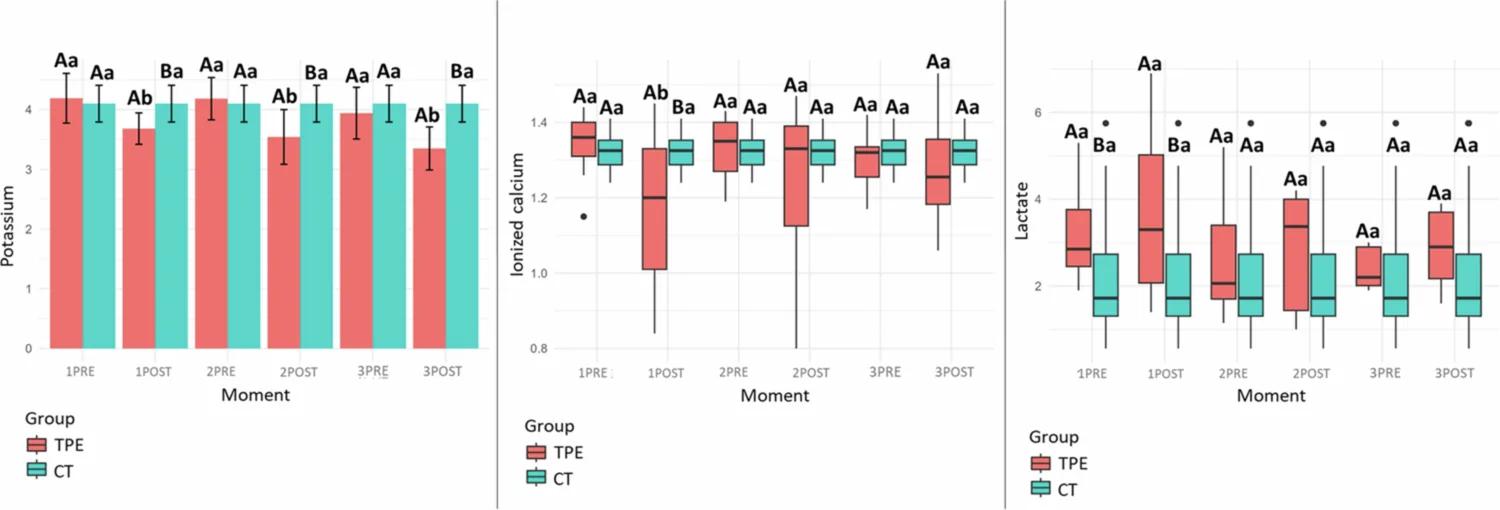
Bar graphs or boxplots of potassium (average), ionised calcium (iCa), and lactate according to group and time point. Different uppercase letters indicate significant differences between groups at each time point; different lowercase letters indicate significant differences between pre- and post-TPE time points within each session in the GTPE. No comparisons were made between different sessions (1, 2, and 3).

The results related to the evaluation of the animals’ coagulation status revealed lower fibrinogen concentrations in the GTPE group than in the GCT group at the post-TPE time points of the first and second sessions. This reduction resulted from a significant decrease in fibrinogen levels in these post-TPE evaluations compared with the corresponding pre-TPE evaluations within the GTPE. Compared with that of the GCT, the prothrombin time (PT) was significantly shorter at the pre-TPE time point in all sessions of the GTPE. However, when comparing the pre- and post-TPE time points within the GTPE, a significant increase in PT was observed after the procedure in all evaluated sessions, resulting in a significantly greater post-TPE value during the third session than in the GCT. No significant differences were detected for the activated partial thromboplastin time (aPTT) between groups or between time points (Figure 4 and Table 2).

**Figure 4.**
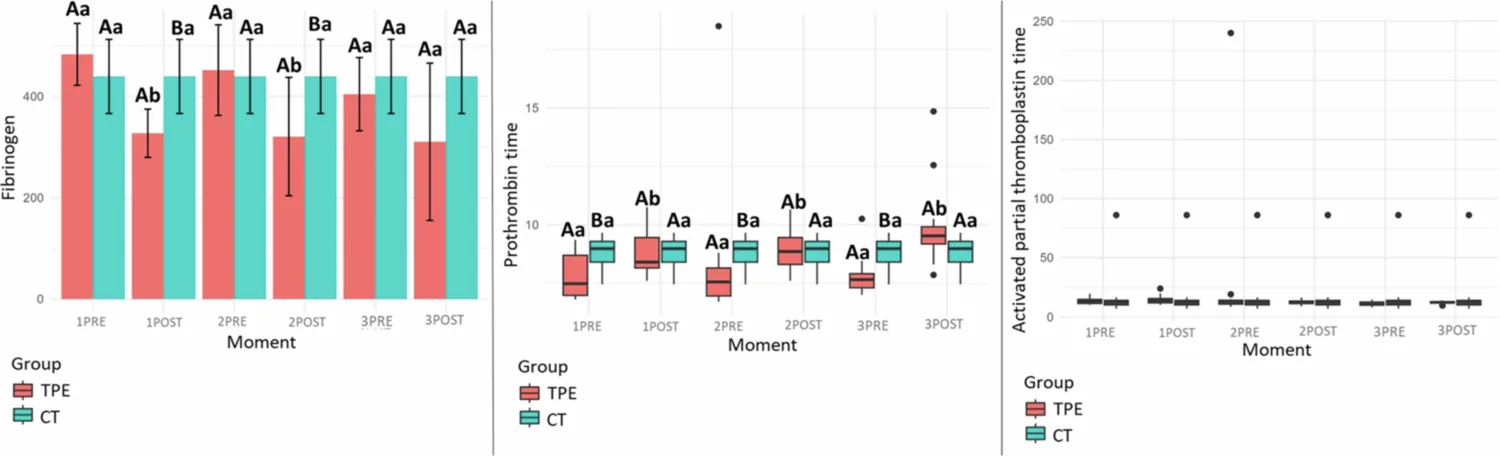
Bar or box plots of fibrinogen concentration (average), prothrombin time (PT), and activated partial thromboplastin time (aPTT). Different uppercase letters indicate significant differences between groups at each time point; different lowercase letters indicate significant differences between time points (pre- and post-) within each session in the GTPE. No comparisons were made between sessions (1, 2, and 3).

**Table 2.** Descriptive and comparative analysis of coagulation variables at pre- and post-procedure time points in the TPE group (GTPE).

		TPE group (GTPE)				Clinical treatment group (GCT)				*p*-value (between groups)
Variable	Time point	M	MD	SD	IQR	M	MD	SD	IQR	
Fibrinogen	1PRE	482.86	473.95	61.02	17.75	439.12	419.02	73.18	80.35	0.103**
	1POST	327.16	320.73	47.61	42.56	439.12	419.02	73.18	80.35	**<0.001** **
	*p*-value	**<0.001** ****					-			
	2PRE	451.74	481.35	89.32	58.40	439.12	419.02	73.18	80.35	0.693**
	2POST	320.57	287.45	116.79	69.13	439.12	419.02	73.18	80.35	**0.010** **
	*p*-value	**0.047** ****					-			
	3PRE	404.12	425.90	72.25	106.20	439.12	419.02	73.18	80.35	0.223**
	3POST	310.34	289.20	155.12	86.25	439.12	419.02	73.18	80.35	0.055**
	*p*-value	-					-			
Prothrombin time (PT)	1PRE	7.76	7.47	0.92	1.72	8.82	8.98	0.64	0.89	**0.005** *
	1POST	8.78	8.40	0.99	1.30	8.82	8.98	0.64	0.89	0.709*
	*p*-value	**0.003** ***					-			
	2PRE	8.34	7.55	3.12	1.20	8.82	8.98	0.64	0.89	**0.002** *
	2POST	8.95	8.85	0.97	1.15	8.82	8.98	0.64	0.89	0.844*
	*p*-value	**0.025** ***					-			
	3PRE	7.81	7.65	0.85	0.60	8.82	8.98	0.64	0.89	**0.001** *
	3POST	9.99	9.52	1.91	0.74	8.82	8.98	0.64	0.89	**0.027** *
	*p*-value	**<0.001** ***					-			
Activated partial thromboplastin time (aPTT)	1PRE	13.47	13.00	2.63	3.40	16.68	12.05	19.35	3.80	0.269*
	1POST	14.57	13.45	3.79	3.65	16.68	12.05	19.35	3.80	0.123*
	*p*-value	0.576***					-			
	2PRE	29.94	12.10	63.17	3.25	16.68	12.05	19.35	3.80	0.678*
	2POST	12.37	12.10	2.02	2.10	16.68	12.05	19.35	3.80	0.892*
	*p*-value	0.666***					-			
	3PRE	11.22	11.30	2.07	2.55	16.68	12.05	19.35	3.80	0.407
	3POST	12.05	12.20	1.36	1.20	16.68	12.05	19.35	3.80	0.922
	*p*-value	0.204***					-			

^*^ Mann–Whitney U test.

^**^ Student’s t-test.

^***^ Wilcoxon test.

^****^ Paired t-test; M = mean; MD = median; SD = standard deviation; IQR = interquartile range.

Clinical results were also recorded subjectively. Although TPE was administered to dogs for a relatively short period, visible clinical improvement was observed in all treated animals (100%), which exhibited documented physical abnormalities (polyarthritis, dermatological lesions, uveitis, and generalised external inflammation) (Figure 5).

**Figure 5.**
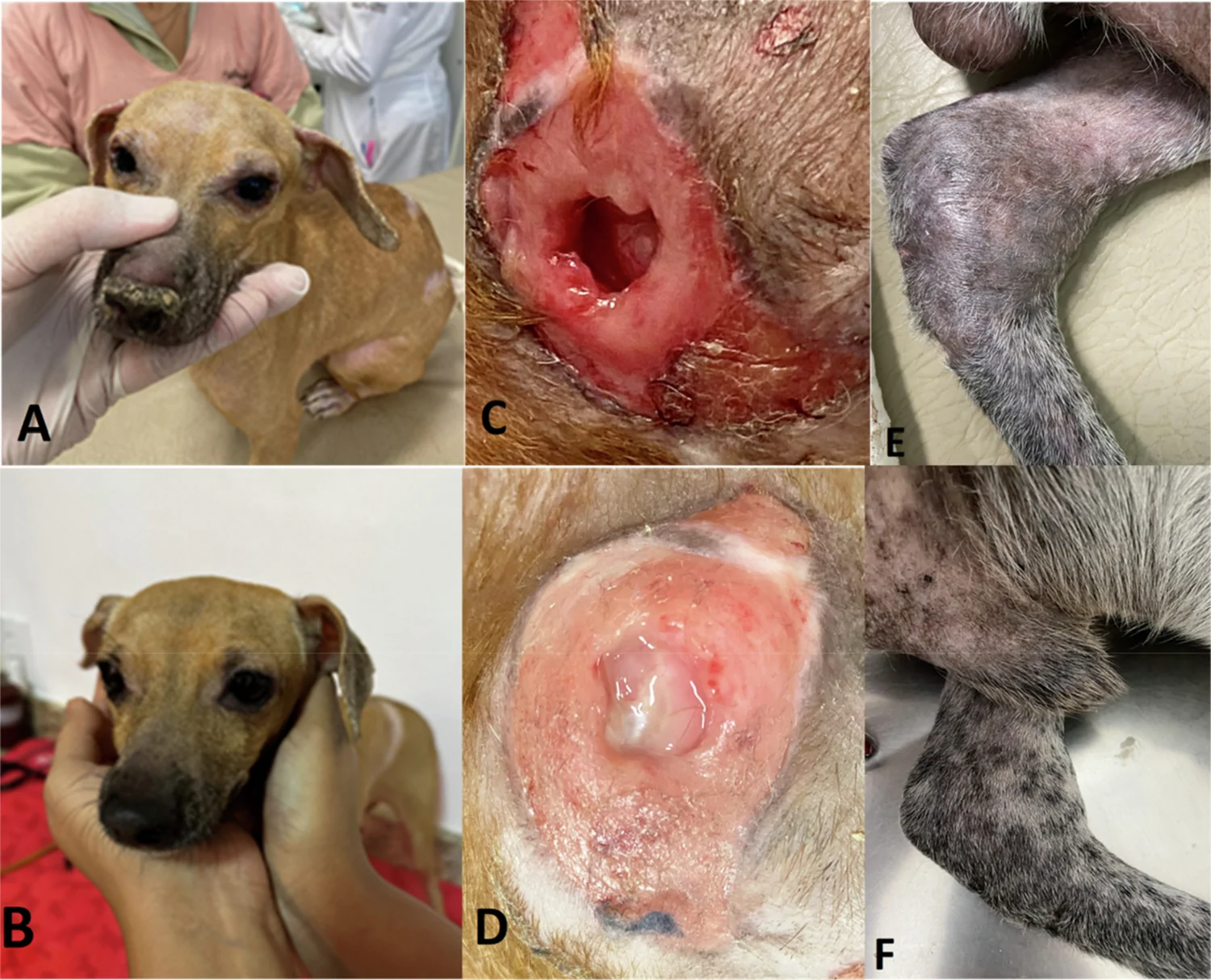
Aspects of inflammatory processes before (A, C, and E) and after (B, D, and F) three consecutive sessions of therapeutic plasma exchange (TPE) applied to dogs with canine visceral leishmaniasis (CVL).

## 4. Discussion

Canine visceral leishmaniasis (CVL) is a chronic systemic parasitic disease associated with marked immune dysregulation and persistent antigenic stimulation, affecting multiple organs and systems (Morales-Yuste et al. 2022; Gazyağci et al. 2023). Chronic exposure to *Leishmania infantum* antigens promotes polyclonal B-cell activation and an intense humoral immune response, leading to hypergammaglobulinemia (Carvalho et al. 2024).

In this context, immune complex formation and systemic inflammation may exacerbate tissue damage and contribute to clinical and laboratory abnormalities (Koenhemsi et al. 2020). Among these abnormalities, anaemia, as observed in the patients in this study, may develop as a consequence of systemic inflammation, extravascular haemolysis, immune-mediated mechanisms, or bone marrow involvement (Manzillo et al. 2006; Almeida et al. 2021).

In the present study, anaemia was observed only in the TPE group, suggesting a more pronounced haematologic impact of the disease in these animals. However, the TPE procedure itself did not influence erythrocyte count, haemoglobin concentration, or haematocrit values. Despite being an extracorporeal procedure, in which some erythrocyte loss may occur owing to retention within the circuit, it is important to emphasise that TPE session prescriptions are designed with haematologic safety in mind, which is particularly relevant for small patients such as children and small animals. This includes the possibility of priming the extracorporeal circuit with packed red blood cells according to the patient’s blood volume, which is estimated on the basis of haematocrit (Sekiguchi et al. 2020; Luzzi et al. 2021; Sever et al. 2023). This safety measure was adopted for the patients in this study and may explain the lack of changes observed between time points. Likewise, the reduction in MCHC and the overall numerical increase in metarubricytes may be associated with the chronicity of the CVL. Nevertheless, the reduction in total protein might be expected as demonstrated by previous studies (Crump and Seshadri 2009; Maia et al. 2025).

Following TPE, a reduction in the platelet count is expected due to the intrinsic properties of this apheresis technique. This phenomenon has been demonstrated in both humans (Tormey et al. 2010; Thölking et al. 2015) and dogs (Francey and Schweighauser 2019). Although the decrease in platelet count in the GTPE of the present study was not statistically significant between the pre- and post-procedure time points, a progressive decline was observed across consecutive sessions, leading to a significant difference compared with the GC from the second TPE session onward. Despite the absence of significant platelet reductions or thrombocytopenia, the progressive decrease in platelet count observed throughout the sessions should be considered, as it may contribute to alterations in haemostatic balance, although no clinically significant haemorrhagic events were detected.

An increase in total leucocytes and segmented neutrophils, along with a decrease in lymphocytes, observed in the post-TPE time points, draws attention to the potential activation of an acute-phase response possibly triggered by the extracorporeal circulation itself, a mechanism also reported in other extracorporeal procedures such as haemodialyses (Picelli de Azevedo et al. 2022). However, it is important to emphasise that the immunomodulatory effects of TPE are not restricted to leucocyte counts. Rather, they extend more prominently to the modulation of immunoglobulins, cytokines, and complement proteins (Reeves and Winters 2014; Scagnelli et al. 2018).

The influence of TPE on blood gas parameters varies across studies (Francey and Schweighauser 2019). Nonetheless, as observed in the present study, a reduction in pH has also been reported by Francey et al. (2021). It is possible that plasma exchange and, consequently, the replacement of circulating components may contribute to this alteration, although this remains inconclusive and may depend on the underlying disease itself. Additionally, considering that dogs in the TPE group presented higher lactate concentrations than those in the clinical treatment group did, one may speculate that the correction or improvement of peripheral hypoperfusion secondary to dehydration during the procedure also contributed to the observed changes.

The reduction in ionised calcium (iCa) observed after the first TPE session in the present study is a well-documented finding among patients undergoing TPE. This condition can be attributed to the use of citrate as an anticoagulant, which chelates ionised calcium to inhibit the coagulation cascade, thereby decreasing circulating iCa levels (Schetz 2001; Crump and Seshadri 2009; Francey and Schweighauser 2019). Nevertheless, continuous intravenous supplementation with 10% calcium gluconate prevented sustained hypocalcemia and prevented its recurrence in subsequent sessions. Even so, clinical signs consistent with transient hypocalcemia were observed in two dogs in this study—an expected but easily reversible complication that must always be anticipated during the procedure, as similarly documented in other reports (Francey and Schweighauser 2019; Kopecny et al. 2020).

On the basis of the coagulation profile results, the reduction in fibrinogen and increase in prothrombin time (PT) observed in this study are consistent with previous reports in humans undergoing therapeutic plasma exchange (Thölking et al. 2015), suggesting that similar haemostatic alterations may also occur in dogs. These findings can be explained by the nonselective nature of TPE, which promotes the removal of both pathological and physiological plasma components, including coagulation factors and regulatory proteins (Kaplan 2013; de Back et al. 2019).

The reduction in fibrinogen levels observed after TPE sessions likely reflects its direct removal during plasma exchange, as well as the dilutional effect associated with replacement fluids. Although fresh frozen plasma (FFP) was used as part of the replacement solution, the proportion administered may not have been sufficient to fully compensate for the loss of coagulation factors, which may explain the observed decrease. In dogs, a marked decrease in fibrinogen concentration has been reported following TPE, even in the absence of relevant changes in other coagulation times (Francey and Schweighauser 2019). Similarly, the increase in PT, in contrast to the stability of activated partial thromboplastin time (aPTT), suggests a differential impact on the extrinsic coagulation pathway. PT is particularly sensitive to changes in coagulation factor VII, which has a short half-life and may be more susceptible to depletion or dilution during the procedure (WFH, 2020). Although conventional coagulation tests were performed, the specific measurement of labile coagulation factors was not included in this study, which represents a limitation.

The replacement of the patient’s plasma with substitution fluids that differ from fresh frozen plasma (FFP) further influences these findings, as such fluids do not provide the coagulation factors removed during the procedure (Kohli et al. 2022). Although the aforementioned alterations were observed even when at least 50% of FFP was used as replacement fluid, it is important to note that all coagulation parameters evaluated remained within the reference range for the species (Gottlieb et al. 2017; Thomason et al. 2018), which may precisely reflect the protective effect of using FFP as part of the replacement solution.

In human medicine, partial plasma replacement is also used in patients already receiving anticoagulant therapy who require TPE, aiming to ensure greater haemostatic safety (Soares Ferreira Júnior et al. 2022). Moreover, even when fibrinogen concentrate is employed as a replacement fluid during TPE, haemostatic parameters tend to indicate a moderately worsened coagulation profile compared to partial FFP replacement (Zrimsek et al. 2024). Conversely, using FFP as the sole replacement fluid during TPE although theoretically ideal is largely discouraged in most cases, since transfusion of a blood component may lead to adverse reactions and negative outcomes, as well as increase the risk of citrate-induced hypocalcemia (Soares Ferreira Júnior et al. 2022). In the present study, a hypersensitivity reaction was observed in only one animal.

Notably, although conventional coagulation assays remain the standard for haemostatic evaluation, they may fail to detect subtle or functional alterations, such as those related to the viscoelastic properties of the clot (Luddington 2005). Human studies assessing haemostasis after TPE have shown that clot formation and firmness, evaluated through rotational thromboelastometry, are also compromised following this procedure (Thölking et al. 2015; Soares Ferreira Júnior et al. 2022; Zrimsek et al. 2024), highlighting an important area for future research in veterinary medicine. Another relevant aspect concerns the possibility that plasma proteins with natural anticoagulant functions may also be removed during TPE (Soares Ferreira Júnior et al. 2022), which could contribute to maintaining a delicate balance between bleeding and thrombosis tendencies. Thus, although aPTT was not significantly altered by TPE and coagulation parameters were maintained within the reference ranges in this study, the potential risk of haemorrhagic or thrombotic events should not be disregarded.

Although the haemostatic alterations induced by TPE are generally considered transient, performing the procedure on consecutive days may represent an additional risk for bleeding, particularly in patients undergoing invasive or surgical interventions (Flaum et al. 1979). In the present study, although none of the animals were subjected to invasive procedures throughout the three consecutive days of TPE, haemorrhagic complications were not observed, except for a single case of pronounced haematuria in one dog. However, this event was most likely related to a transfusion reaction, corresponding to an isolated and rapidly resolved episode. Therefore, within the conditions of this study, the three-day consecutive TPE protocol appeared to be hemostatically well tolerated and immunologically effective in the absence of surgical interventions during the course of apheresis treatment. Although the primary interpretation of the effects of TPE on haemostasis in this study is based mainly on intraindividual comparisons (before and after the procedure), age differences between groups should be interpreted with caution. Likewise, although age is not expected to be a major determinant of conventional coagulation times, such as PT and aPTT, these differences may reflect underlying clinical heterogeneity between groups and therefore represent a limitation of between-group comparability.

Besides the two dogs that developed the aforementioned complications, all the remaining animals maintained clinical stability, and their vital parameters remained within the expected physiological range throughout the TPE sessions. The maintenance of physical parameters within the species’ reference interval, along with the low frequency of mild, promptly manageable clinical complications, has also been reported in other studies conducted in dogs (Francey and Schweighauser 2019; Kopecny et al. 2020), supporting the safety of TPE when specific priming solutions, appropriate replacement fluids, and calcium supplementation are properly applied.

Although clinically relevant complications were limited, it is important to emphasise that other mild and transient adverse events, such as hypocalcemia and vomiting were observed. These findings suggest that although the procedure was well tolerated in most cases, it is not entirely free of effects that require monitoring. Given the relatively small sample size, the frequency of adverse events should be interpreted with caution, and studies including a larger number of animals are needed to better establish the safety profile of TPE in dogs with CVL.

Furthermore, the marked clinical improvement in the physical manifestations associated with the zoonosis, combined with the removal of potentially immunogenic proteins, underscores the therapeutic potential of this apheresis modality and its valuable contribution in cases refractory to conventional treatment.

## 5. Conclusion

In conclusion, centrifugation-based therapeutic plasma exchange in dogs with canine visceral leishmaniasis was associated with mild and predominantly transient laboratory changes in coagulation parameters that remained within reference ranges and were not accompanied by clinically significant bleeding events. Under the conditions of this study, TPE appeared to be generally well tolerated when appropriate replacement and supportive protocols were applied, and clinical improvement was observed in treated animals. Further studies are needed to better define its long-term safety profile and therapeutic impact.

## Supplementary Material

Supplementary_Table_2clean.docx

Supplementary_Table_3clean.docx Supplementary_Table_3clean.docx

Supplementary_Table_1_with_clean.docx Supplementary_Table_1_with_clean.docx
