# Supplementary material for: Effects of centrifugation-based therapeutic plasma exchange on coagulation parameters in dogs with canine visceral leishmaniasis
Source: Vet Q. 2026 Oct 14;46(1):2706032. doi: 10.1080/01652176.2026.2706032 (PMC13670271; doi:10.1080/01652176.2026.2706032)
Supplement: Supplementary file 1 [file TVEQ_A_2706032_SM9434.docx]

| Variable | Session | Pre-TPE | | | | Post-TPE | | | | p-value  (between time points per session) |
| --- | --- | --- | --- | --- | --- | --- | --- | --- | --- | --- |
|  |  | **M** | **MD** | **SD** | **IQR** | **M** | **MD** | **SD** | **IQR** |  |
| MCV (fL) | 1 | 70.57 | 67.70 | 7.33 | 9.80 | 69.99 | 68.90 | 4.34 | 6.90 | 0.661** |
|  | 2 | 71.10 | 70.40 | 5.81 | 6.60 | 71.27 | 70.80 | 5.64 | 4.70 | 0.785** |
|  | 3 | 70.21 | 70.30 | 5.27 | 5.60 | 70.19 | 70.05 | 4.06 | 4.40 | 0.657** |
| MCHC (g/dL) | 1 | 32.68 | 32.60 | 1.39 | 2.10 | 32.52 | 32.50 | 1.54 | 1.80 | 0.729** |
|  | 2 | 31.76 | 31.60 | 1.37 | 1.60 | 31.99 | 32.30 | 2.14 | 1.60 | 0.624* |
|  | 3 | 33.42 | 33.30 | 1.48 | 1.10 | 32.90 | 32.30 | 1.55 | 2.07 | **0.005**** |
| Total protein (g/dL) | 1 | 9.07 | 8.50 | 1.43 | 1.60 | 5.31 | 5.40 | 0.57 | 0.80 | **<0.001**** |
|  | 2 | 6.63 | 6.20 | 0.96 | 1.20 | 4.29 | 4.20 | 0.55 | 0.80 | **<0.001**** |
|  | 3 | 6.15 | 6.00 | 0.81 | 0.80 | 4.17 | 4.10 | 0.31 | 0.25 | **<0.001**** |
| Metarubricytes (/100 WBC) | 1 | 0.46 | 0.00 | 1.13 | 0.00 | 2.08 | 0.00 | 5.81 | 0.00 | 0.750* |
|  | 2 | 1.38 | 0.00 | 3.59 | 1.00 | 1.31 | 0.00 | 4.71 | 0.00 | 0.750* |
|  | 3 | 1.15 | 0.00 | 3.60 | 0.00 | 4.00 | 0.00 | 12.62 | 0.50 | 0.500* |
| RDW (%) | 1 | 8.98 | 13.20 | 7.52 | 14.20 | 5.45 | 0.00 | 7.19 | 13.70 | 0.359* |
|  | 2 | 5.44 | 0.00 | 7.24 | 13.10 | 7.52 | 12.60 | 7.28 | 13.60 | 0.496* |
|  | 3 | 7.74 | 12.50 | 7.37 | 13.80 | 8.14 | 12.65 | 7.24 | 14.05 | 1.000* |
| *Wilcoxon signed-rank; **paired t-test | | | | | | | | | | |
| M = mean; MD = median; SD = standard deviation; IQR = interquartile range. | | | | | | | | | | |
| Session 3 / animal 9 with no data at the post-TPT; n=12 in this session. | | | | | | | | | | |

**Supplementary Table 1.** Descriptive and comparative analysis of MVC, MCHC, total protein, metarubricytes, and RDW at pre- and post-procedure time points in the TPE group (GTPE).
