# Supplementary material for: Effects of centrifugation-based therapeutic plasma exchange on coagulation parameters in dogs with canine visceral leishmaniasis
Source: Vet Q. 2026 Oct 14;46(1):2706032. doi: 10.1080/01652176.2026.2706032 (PMC13670271; doi:10.1080/01652176.2026.2706032)
Supplement: Supplementary file 2 [file TVEQ_A_2706032_SM9435.docx]

**Detailed Statistical Results**

**Supplementary Table 3.** Descriptive statistics of blood gases and electrolytes variables according to group and time points.

|  |  | **TPE Group (GTPE)** | | | | **Clinical Treatment Group (GCT)** | | | | **p-value**  **(between groups)** |
| --- | --- | --- | --- | --- | --- | --- | --- | --- | --- | --- |
| **Variable** | **Time Point** | **M** | **MD** | **SD** | **IQR** | **M** | **MD** | **SD** | **IQR** |  |
| pH | 1PRE | 7.35 | 7.36 | 0.03 | 0.04 | 7.39 | 7.38 | 0.05 | 0.01 | <0.001* |
|  | 1POST | 7.27 | 7.27 | 0.05 | 0.05 | 7.39 | 7.38 | 0.05 | 0.01 | <0.001* |
|  | p-value | 0.002*** | | | |  | - |  |  |  |
|  | 2PRE | 7.36 | 7.37 | 0.04 | 0.04 | 7.39 | 7.38 | 0.05 | 0.01 | 0.066* |
|  | 2POST | 7.31 | 7.31 | 0.04 | 0.03 | 7.39 | 7.38 | 0.05 | 0.01 | <0.001* |
|  | p-value | 0.004*** | | | |  | - |  |  |  |
|  | 3PRE | 7.38 | 7.38 | 0.03 | 0.04 | 7.39 | 7.38 | 0.05 | 0.01 | 0.566* |
|  | 3POST | 7.32 | 7.32 | 0.04 | 0.05 | 7.39 | 7.38 | 0.05 | 0.01 | <0.001* |
|  | p-value | 0.006*** | | | |  | - |  |  |  |
| BEecf | 1PRE | -4.03 | -4.40 | 2.47 | 2.50 | -3.97 | -3.45 | 2.55 | 4.03 | 0.948** |
|  | 1POST | -8.70 | -8.20 | 1.93 | 3.00 | -3.97 | -3.45 | 2.55 | 4.03 | <0.001** |
|  | p-value | <0.001**** | | | |  | - |  |  |  |
|  | 2PRE | -2.36 | -2.90 | 2.53 | 4.10 | -3.97 | -3.45 | 2.55 | 4.03 | 0.102** |
|  | 2POST | -6.75 | -6.70 | 1.75 | 1.50 | -3.97 | -3.45 | 2.55 | 4.03 | 0.002** |
|  | p-value | <0.001**** | | | |  | - |  |  |  |
|  | 3PRE | -0.89 | -0.65 | 1.35 | 1.82 | -3.97 | -3.45 | 2.55 | 4.03 | <0.001** |
|  | 3POST | -5.73 | -5.60 | 1.26 | 1.88 | -3.97 | -3.45 | 2.55 | 4.03 | 0.028** |
|  | p-value | <0.001**** | | | |  | - |  |  |  |
| HCO₃⁻ | 1PRE | 20.55 | 20.80 | 2.46 | 2.80 | 20.81 | 21.00 | 2.41 | 3.05 | 0.772** |
|  | 1POST | 16.88 | 17.40 | 2.04 | 2.30 | 20.81 | 21.00 | 2.41 | 3.05 | <0.001** |
|  | p-value | <0.001**** | | | |  | - |  |  |  |
|  | 2PRE | 22.13 | 22.40 | 2.52 | 4.10 | 20.81 | 21.00 | 2.41 | 3.05 | 0.166** |
|  | 2POST | 18.47 | 18.30 | 2.19 | 2.50 | 20.81 | 21.00 | 2.41 | 3.05 | 0.015** |
|  | p-value | <0.001**** | | | |  | - |  |  |  |
|  | 3PRE | 23.45 | 23.60 | 1.33 | 1.85 | 20.81 | 21.00 | 2.41 | 3.05 | 0.001** |
|  | 3POST | 19.16 | 19.10 | 1.20 | 1.23 | 20.81 | 21.00 | 2.41 | 3.05 | 0.030** |
|  | p-value | <0.001**** | | | |  | - |  |  |  |
| Chloride | 1PRE | 113.25 | 112.50 | 6.06 | 4.75 | 119.19 | 119.00 | 2.20 | 2.25 | 0.006** |
|  | 1POST | 111.77 | 111.00 | 4.87 | 3.00 | 119.19 | 119.00 | 2.20 | 2.25 | <0.001** |
|  | p-value | 0.018**** | | | |  | - |  |  |  |
|  | 2PRE | 113.42 | 112.50 | 4.40 | 3.50 | 119.19 | 119.00 | 2.20 | 2.25 | <0.001** |
|  | 2POST | 111.30 | 111.00 | 4.42 | 2.00 | 119.19 | 119.00 | 2.20 | 2.25 | <0.001** |
|  | p-value | 0.042**** | | | |  | - |  |  |  |
|  | 3PRE | 111.64 | 111.00 | 5.39 | 5.00 | 119.19 | 119.00 | 2.20 | 2.25 | <0.001** |
|  | 3POST | 108.90 | 109.00 | 6.76 | 6.00 | 119.19 | 119.00 | 2.20 | 2.25 | <0.001** |
|  | p-value | 0.141**** | | | |  | - |  |  |  |
| Potassium | 1PRE | 4.19 | 4.30 | 0.42 | 0.53 | 4.10 | 4.00 | 0.31 | 0.42 | 0.520** |
|  | 1POST | 3.68 | 3.71 | 0.26 | 0.16 | 4.10 | 4.00 | 0.31 | 0.42 | <0.001** |
|  | p-value | 0.003**** | | | |  | - |  |  |  |
|  | 2PRE | 4.18 | 4.11 | 0.35 | 0.50 | 4.10 | 4.00 | 0.31 | 0.42 | 0.512** |
|  | 2POST | 3.54 | 3.62 | 0.46 | 0.56 | 4.10 | 4.00 | 0.31 | 0.42 | 0.003** |
|  | p-value | 0.001**** | | | |  | - |  |  |  |
|  | 3PRE | 3.94 | 4.10 | 0.43 | 0.62 | 4.10 | 4.00 | 0.31 | 0.42 | 0.306** |
|  | 3POST | 3.35 | 3.40 | 0.36 | 0.49 | 4.10 | 4.00 | 0.31 | 0.42 | <0.001** |
|  | p-value | 0.001**** | | | |  | - |  |  |  |
| Sodium | 1PRE | 150.85 | 149.00 | 6.14 | 8.00 | 147.19 | 147.50 | 1.56 | 2.00 | 0.073* |
|  | 1POST | 150.62 | 149.00 | 7.15 | 5.00 | 147.19 | 147.50 | 1.56 | 2.00 | 0.049* |
|  | p-value | 0.687*** | | | |  | - |  |  |  |
|  | 2PRE | 151.54 | 151.00 | 5.56 | 4.00 | 147.19 | 147.50 | 1.56 | 2.00 | 0.002* |
|  | 2POST | 149.64 | 149.00 | 6.89 | 5.50 | 147.19 | 147.50 | 1.56 | 2.00 | 0.357* |
|  | p-value | 0.537*** | | | |  |  |  |  |  |
|  | 3PRE | 150.36 | 149.00 | 7.31 | 3.50 | 147.19 | 147.50 | 1.56 | 2.00 | 0.105* |
|  | 3POST | 149.00 | 148.00 | 7.69 | 5.75 | 147.19 | 147.50 | 1.56 | 2.00 | 0.788* |
|  | p-value | 0.756*** | | | |  | - |  |  |  |
| Ionized calcium | 1PRE | 1.34 | 1.36 | 0.08 | 0.09 | 1.32 | 1.33 | 0.05 | 0.06 | 0.165* |
|  | 1POST | 1.18 | 1.20 | 0.19 | 0.32 | 1.32 | 1.33 | 0.05 | 0.06 | 0.043* |
|  | p-value | 0.008*** | | | |  | - |  |  |  |
|  | 2PRE | 1.33 | 1.35 | 0.08 | 0.13 | 1.32 | 1.33 | 0.05 | 0.06 | 0.598* |
|  | 2POST | 1.23 | 1.33 | 0.23 | 0.27 | 1.32 | 1.33 | 0.05 | 0.06 | 0.675* |
|  | p-value | 0.240*** | | | |  | - |  |  |  |
|  | 3PRE | 1.30 | 1.32 | 0.07 | 0.08 | 1.32 | 1.33 | 0.05 | 0.06 | 0.428* |
|  | 3POST | 1.27 | 1.25 | 0.16 | 0.17 | 1.32 | 1.33 | 0.05 | 0.06 | 0.291* |
|  | p-value | 0.375*** | | | |  | - |  |  |  |
| Lactate | 1PRE | 3.19 | 2.85 | 1.13 | 1.31 | 2.15 | 1.72 | 1.46 | 1.42 | 0.035* |
|  | 1POST | 3.75 | 3.30 | 1.84 | 2.95 | 2.15 | 1.72 | 1.46 | 1.42 | 0.023* |
|  | p-value | - | | | |  | - |  |  |  |
|  | 2PRE | 2.59 | 2.06 | 1.36 | 1.70 | 2.15 | 1.72 | 1.46 | 1.42 | 0.350* |
|  | 2POST | 2.78 | 3.37 | 1.42 | 2.56 | 2.15 | 1.72 | 1.46 | 1.42 | 0.332* |
|  | p-value | - | | | |  | - |  |  |  |
|  | 3PRE | 2.40 | 2.20 | 0.51 | 0.89 | 2.15 | 1.72 | 1.46 | 1.42 | 0.200* |
|  | 3POST | 2.85 | 2.90 | 0.98 | 1.53 | 2.15 | 1.72 | 1.46 | 1.42 | 0.148* |
|  | p-value | 0.625*** | | | |  | - |  |  |  |
| *Mann–Whitney U test; **Student’s t-test; ***Wilcoxon test; ****Paired t-test; M = mean; MD = median; SD = standard deviation; IQR = interquartile range. | | | | | | | | | | |
