# Supplementary material for: Effects of centrifugation-based therapeutic plasma exchange on coagulation parameters in dogs with canine visceral leishmaniasis
Source: Vet Q. 2026 Oct 14;46(1):2706032. doi: 10.1080/01652176.2026.2706032 (PMC13670271; doi:10.1080/01652176.2026.2706032)
Supplement: Supplementary file 3 [file TVEQ_A_2706032_SM9436.docx]

**Detailed Statistical Results**

**Supplementary Table 2.** Descriptive statistics of hematological variables according to group and time points.

|  |  | **TPE Group (GTPE)** | | | | **Clinical treatment group (GCT)** | | | | **p-value**  **(between groups)** | |
| --- | --- | --- | --- | --- | --- | --- | --- | --- | --- | --- | --- |
| **Variable** | **Time Point** | **M** | **MD** | **SD** | **IQR** | **M** | **MD** | **SD** | **IQR** |  |  |
| Red blood cells (RBC) | 1PRE | 4.04 | 3.89 | 1.49 | 1.12 | 6.70 | 6.81 | 0.94 | 1.25 | | <0.001 |
|  | 1POST | 4.20 | 4.27 | 1.57 | 1.90 | 6.70 | 6.81 | 0.94 | 1.25 | | <0.001 |
|  | p-value** | 0.328 | | | |  | - |  |  | |  |
|  | 2PRE | 3.61 | 3.71 | 1.23 | 1.55 | 6.70 | 6.81 | 0.94 | 1.25 | | <0.001 |
|  | 2POST | 3.78 | 3.69 | 1.38 | 1.80 | 6.70 | 6.81 | 0.94 | 1.25 | | <0.001 |
|  | p-value | 0.168 | | | |  | - |  |  | |  |
|  | 3PRE | 3.66 | 3.46 | 1.17 | 1.03 | 6.70 | 6.81 | 0.94 | 1.25 | | <0.001 |
|  | 3POST | 3.50 | 3.40 | 1.00 | 1.13 | 6.70 | 6.81 | 0.94 | 1.25 | | <0.001 |
|  | p-value** | 0.505 | | | |  | - |  |  | |  |
| Hemoglobin | 1PRE | 9.18 | 9.4 | 3.16 | 2.2 | 14.74 | 15.30 | 2.35 | 3.80 | | <0.001 |
|  | 1POST | 9.44 | 10.00 | 3.20 | 3.9 | 14.74 | 15.30 | 2.35 | 3.80 | | <0.001 |
|  | p-value** | 0.382 | | | |  | - |  |  | |  |
|  | 2PRE | 8.10 | 8.50 | 2.68 | 3.2 | 14.74 | 15.30 | 2.35 | 3.80 | | <0.001 |
|  | 2POST | 8.63 | 8.40 | 3.16 | 4.9 | 14.74 | 15.30 | 2.35 | 3.80 | | <0.001 |
|  | p-value** | 0.124 | | | |  | - |  |  | |  |
|  | 3PRE | 8.52 | 7.70 | 2.66 | 2.20 | 14.74 | 15.30 | 2.35 | 3.80 | | <0.001 |
|  | 3POST | 8.04 | 7.60 | 2.32 | 2.47 | 14.74 | 15.30 | 2.35 | 3.80 | | <0.001 |
|  | p-value** | 0.247 | | | |  | - |  |  | |  |
| Hematocrit | 1PRE | 27.92 | 27.00 | 9.20 | 7.00 | 42.21 | 43.40 | 6.55 | 9.92 | | <0.001 |
|  | 1POST | 29.15 | 32.00 | 10.12 | 13.00 | 42.21 | 43.40 | 6.55 | 9.92 | | 0.001 |
|  | p-value** | 0.238 | | | |  | - |  |  | |  |
|  | 2PRE | 25.31 | 26.00 | 7.83 | 9.00 | 42.21 | 43.40 | 6.55 | 9.92 | | <0.001 |
|  | 2POST | 26.69 | 26.00 | 8.95 | 14.00 | 42.21 | 43.40 | 6.55 | 9.92 | | <0.001 |
|  | p-value** | 0.081 | | | |  | - |  |  | |  |
|  | 3PRE | 25.54 | 24.00 | 7.82 | 9.00 | 42.21 | 43.40 | 6.55 | 9.92 | | <0.001 |
|  | 3POST | 24.42 | 23.50 | 6.60 | 8.50 | 42.21 | 43.40 | 6.55 | 9.92 | | <0.001 |
|  | p-value** | 0.681 | | | |  | - |  |  | |  |
| Platelets | 1PRE | 234875.00 | 200000.00 | 174444.78 | 74750.00 | 170833.33 | 165000.00 | 58347.09 | 82250.00 | | 0.279 |
|  | 1POST | 200000.00 | 188000.00 | 74759.61 | 133000.00 | 170833.33 | 165000.00 | 58347.09 | 82250.00 | | 0.587 |
|  | p-value** | - | | | |  | - |  |  | |  |
|  | 2PRE | 144800.00 | 141000.00 | 35786.87 | 19000.00 | 170833.33 | 165000.00 | 58347.09 | 82250.00 | | 0.538 |
|  | 2POST | 66696.25 | 79000.00 | 37076.11 | 37000.00 | 170833.33 | 165000.00 | 58347.09 | 82250.00 | | <0.001 |
|  | p-value** | - | | | |  | - |  |  | |  |
|  | 3PRE | 112500.00 | 134000.00 | 50338.85 | 67750.00 | 170833.33 | 165000.00 | 58347.09 | 82250.00 | | 0.067 |
|  | 3POST | 85142.86 | 81000.00 | 28251.42 | 45000.00 | 170833.33 | 165000.00 | 58347.09 | 82250.00 | | <0.001 |
|  | p-value** | - | | | |  | - |  |  | |  |
| Leukocytes | 1PRE | 10806.15 | 8740.00 | 5016.50 | 3700.00 | 9196.11 | 8870.00 | 2539.19 | 3627.50 | | 0.679 |
|  | 1POST | 21127.69 | 18700.00 | 11246.52 | 18800.00 | 9196.11 | 8870.00 | 2539.19 | 3627.50 | | <0.001 |
|  | p-value** | 0.011 | | | |  | - |  |  | |  |
|  | 2PRE | 11336.15 | 10300.00 | 3819.79 | 2380.00 | 9196.11 | 8870.00 | 2539.19 | 3627.50 | | 0.183 |
|  | 2POST | 17941.67 | 14450.00 | 8705.53 | 9900.00 | 9196.11 | 8870.00 | 2539.19 | 3627.50 | | <0.001 |
|  | p-value** | 0.003 | | | |  | - |  |  | |  |
|  | 3PRE | 12951.54 | 11500.00 | 6474.50 | 2300.00 | 9196.11 | 8870.00 | 2539.19 | 3627.50 | | 0.069 |
|  | 3POST | 20890.83 | 20300.00 | 11588.60 | 12107.50 | 9196.11 | 8870.00 | 2539.19 | 3627.50 | | <0.001 |
|  | p-value** | 0.009 | | | |  | - |  |  | |  |
| Segmented neutrophils | 1PRE | 8744.85 | 6806.00 | 4288.08 | 5144.00 | 5545.56 | 5130.00 | 1847.66 | 1805.00 | | 0.012 |
|  | 1POST | 19282.62 | 16456.00 | 10732.23 | 16643.00 | 5545.56 | 5130.00 | 1847.66 | 1805.00 | | <0.001 |
|  | p-value** | 0.003 | | | |  | - |  |  | |  |
|  | 2PRE | 9134.77 | 8568.00 | 4156.46 | 3890.00 | 5545.56 | 5130.00 | 1847.66 | 1805.00 | | 0.006 |
|  | 2POST | 15395.08 | 12320.00 | 8512.77 | 8019.00 | 5545.56 | 5130.00 | 1847.66 | 1805.00 | | <0.001 |
|  | p-value** | 0.001 | | | |  | - |  |  | |  |
|  | 3PRE | 10488.08 | 9317.00 | 6406.95 | 3368.00 | 5545.56 | 5130.00 | 1847.66 | 1805.00 | | 0.004 |
|  | 3POST | 19028.58 | 18308.00 | 11135.67 | 10347.25 | 5545.56 | 5130.00 | 1847.66 | 1805.00 | | <0.001 |
|  | p-value** | <0.001 | | | |  | - |  |  | |  |
| Lymphocytes | 1PRE | 681.23 | 656.00 | 399.63 | 490.00 | 2394.44 | 2015.00 | 1083.52 | 1370.00 | | <0.001 |
|  | 1POST | 525.46 | 418.00 | 326.65 | 459.00 | 2394.44 | 2015.00 | 1083.52 | 1370.00 | | <0.001 |
|  | p-value** | 0.147 | | | |  | - |  |  | |  |
|  | 2PRE | 846.00 | 714.00 | 814.82 | 612.00 | 2394.44 | 2015.00 | 1083.52 | 1370.00 | | <0.001 |
|  | 2POST | 441.77 | 381.00 | 273.95 | 313.00 | 2394.44 | 2015.00 | 1083.52 | 1370.00 | | <0.001 |
|  | p-value** | 0.021 | | | |  | - |  |  | |  |
|  | 3PRE | 935.08 | 1089.00 | 622.67 | 995.00 | 2394.44 | 2015.00 | 1083.52 | 1370.00 | | <0.001 |
|  | 3POST | 581.42 | 538.50 | 232.11 | 213.00 | 2394.44 | 2015.00 | 1083.52 | 1370.00 | | <0.001 |
|  | p-value** | 0.034 | | | |  | - |  |  | |  |
| Eosinophils | 1PRE | 419.62 | 428.00 | 296.55 | 341.00 | 361.11 | 280.00 | 345.56 | 262.50 | | 0.420 |
|  | 1POST | 328.60 | 317.00 | 167.56 | 150.00 | 361.11 | 280.00 | 345.56 | 262.50 | | 0.682 |
|  | p-value** | - | | | |  | - |  |  | |  |
|  | 2PRE | 277.25 | 284.00 | 164.64 | 127.75 | 361.11 | 280.00 | 345.56 | 262.50 | | 0.803 |
|  | 2POST | 356.83 | 335.00 | 92.92 | 122.75 | 361.11 | 280.00 | 345.56 | 262.50 | | 0.405 |
|  | p-value** | - | | | |  | - |  |  | |  |
|  | 3PRE | 589.86 | 460.00 | 463.66 | 433.00 | 361.11 | 280.00 | 345.56 | 262.50 | | 0.155 |
|  | 3POST | 246.80 | 192.00 | 165.05 | 215.00 | 361.11 | 280.00 | 345.56 | 262.50 | | 0.628 |
|  | p-value** | - | | | |  | - |  |  | |  |
| Monocytes | 1PRE | 1116.92 | 492.00 | 1077.25 | 664.00 | 843.89 | 805.00 | 254.49 | 257.50 | | 0.483 |
|  | 1POST | 1198.92 | 1088.00 | 673.82 | 1049.00 | 843.89 | 805.00 | 254.49 | 257.50 | | 0.207 |
|  | p-value** | 0.588 | | | |  | - |  |  | |  |
|  | 2PRE | 1165.92 | 1051.00 | 630.05 | 841.00 | 843.89 | 805.00 | 254.49 | 257.50 | | 0.173 |
|  | 2POST | 1279.77 | 1340.00 | 740.52 | 1327.00 | 843.89 | 805.00 | 254.49 | 257.50 | | 0.173 |
|  | p-value** | 0.636 | | | |  | - |  |  | |  |
|  | 3PRE | 1297.75 | 1188.50 | 556.27 | 726.25 | 843.89 | 805.00 | 254.49 | 257.50 | | 0.015 |
|  | 3POST | 1158.92 | 914.00 | 907.93 | 1302.5 | 843.89 | 805.00 | 254.49 | 257.50 | | 0.751 |
|  | p-value** | 0.724 | | | |  | - |  |  | |  |
| *Mann–Whitney U test; **Wilcoxon test; M = mean; MD = median; SD = standard deviation; IQR = interquartile range. | | | | | | | | | | | |
